# A Coupled Model of the Cardiovascular and Immune Systems to Analyze the Effects of COVID-19 Infection

**DOI:** 10.3390/biotech14010019

**Published:** 2025-03-12

**Authors:** Camila Ribeiro Passos, Alexandre Altamir Moreira, Ruy Freitas Reis, Rodrigo Weber dos Santos, Marcelo Lobosco, Bernardo Martins Rocha

**Affiliations:** 1 Computational Engineering, Federal University of Juiz de Fora, Juiz de Fora 36036-900, Brazil; camila.passos@estudante.ufjf.br; 2 Mechanical Engineering, Federal University of Juiz de Fora, Juiz de Fora 36036-900, Brazil; alexandre.moreira@estudante.ufjf.br; 3 Graduate Program in Computational Modeling, Federal University of Juiz de Fora, Juiz de Fora 36036-900, Brazil; ruy.reis@ufjf.br (R.F.R.); marcelo.lobosco@ufjf.br (M.L.); 4 Computer Science Department, Federal University of Juiz de Fora, Juiz de Fora 36036-900, Brazil

**Keywords:** COVID-19, viral infection, cardiovascular system, immune system, mathematical models

## Abstract

The COVID-19 pandemic has underscored the importance of understanding the interplay between the cardiovascular and immune systems during viral infections. SARS-CoV-2 enters human cells via the ACE-2 enzyme, initiating a cascade of immune responses. This study presents a coupled mathematical model that integrates the cardiovascular system (CVS) and immune system (IS), capturing their complex interactions during infection. The CVS model, based on ordinary differential equations, describes heart dynamics and pulmonary and systemic circulation, while the IS model simulates immune responses to SARS-CoV-2, including immune cell interactions and cytokine production. A coupling strategy transfers information from the IS to the CVS at specific intervals, enabling the exploration of immune-driven cardiovascular effects. Numerical simulations examined how these interactions influence infection severity and recovery. The coupled model accurately replicated the evolution of cardiac function in survivors and non-survivors of COVID-19. Survivors exhibited a left ventricular ejection fraction (LVEF) reduction of up to 25% while remaining within normal limits, whereas non-survivors showed a severe 4-fold decline, indicative of myocardial dysfunction. Similarly, the right ventricular ejection fraction (RV EF) decreased by approximately 50% in survivors but underwent a drastic 5-fold reduction in non-survivors. These findings highlight the model’s capacity to distinguish differential cardiac dysfunction across clinical outcomes and its potential to enhance our understanding of COVID-19 pathophysiology.

## 1. Introduction

First reported in December 2019, severe acute respiratory syndrome, also known as COVID-19, was responsible for triggering a global pandemic that shook public health and society on an unprecedented scale. Identified in the city of Wuhan, in the province of Hubei, China, the disease quickly spread worldwide, causing the World Health Organization to declare a pandemic on 11 March 2020 [1].

SARS-CoV-2 (*severe acute respiratory syndrome-associated coronavirus 2*), a pathogen associated with the disease is a *Sarbecovirus* (betacoronavirus lineage B) that is used as a receptor for entry into the cell enzyme angiotensin type 2 (ACE-2), a molecule widely present mainly on the surface of cells of the endothelium, myocardium, kidneys, gastrointestinal tract and lungs [2]. Although its function is not entirely known, this molecule is usually related to the regulation of the renin–angiotensin–aldosterone system, which controls blood pressure and fluid balance in the body [2].

COVID-19 has been linked to a range of cardiovascular complications, many of which significantly contribute to the disease’s morbidity and mortality. These complications include myocarditis, arrhythmia, acute coronary syndromes and thromboembolic events [3]. The mechanisms underlying these effects are multi-factorial and include direct viral invasion of cardiac tissue, systemic inflammation, a dysregulated immune response and endothelial dysfunction [4,5]. Furthermore, the hypercoagulable state observed in severe COVID-19 cases has been implicated in the development of venous thromboembolism and other vascular complications [6]. Understanding these cardiovascular implications is critical for effective management and highlights the intricate interplay between the cardiovascular and immune systems during infection.

The interaction between the cardiovascular system and the immune system has been the subject of study due to its importance in understanding health complications [7]. Mathematical and computational models have been widely used to better understand various pathologies, as well as to propose new therapies and treatments. In this context, a coupled model between the cardiovascular and immune systems presents itself as a promising tool for understanding the dynamics between these systems and their implications. In particular, the prevalence of COVID-19 and its effects to date has highlighted the need to understand how these systems interact during an acute viral infection.

The objective of the present work is to present the coupling between mathematical models of the cardiovascular system and the immune system in a scenario of viral infection by COVID-19. Both models are represented by systems of ordinary differential equations (ODEs) and are based on previously published models validated with experimental data. Coupling is done through the imposition of the effects of the viral infection and the subsequent immune response on the cardiovascular system. Computational simulations for different scenarios are presented, and the results are analyzed based on reference values from the literature for normal and pathological conditions.

## 2. Mathematical Models

Next, the mathematical modeling used to represent the cardiovascular system is described through a compartment model involving the cardiac chambers and the pulmonary and systemic circulation, as well as the immune system model. Subsequently, it describes how the effects of COVID-19 infection are applied to the cardiovascular model based on changes in some parameters. Finally, the strategy used to couple the immunological model is presented to characterize the effects of COVID-19, the response of this system during infection and its effects on the cardiovascular system.

### 2.1. Cardiovascular Model

The cardiovascular system (CVS) model used is based on ordinary differential equations and was originally proposed by Colunga et al. [8]. This model of the cardiovascular system is based on eight compartments and is capable of representing the characteristics of cardiac, systemic and pulmonary circulation, specifically in terms of pressure (mmHg), flow (mL/s) and volume (mL). The structure of the model is formulated with analogies to electrical circuits, in which blood pressure is equivalent to voltage, flow to current, volume to electrical charge and compliance to capacitance, as explained in the literature [9,10].

**Figure 1:**
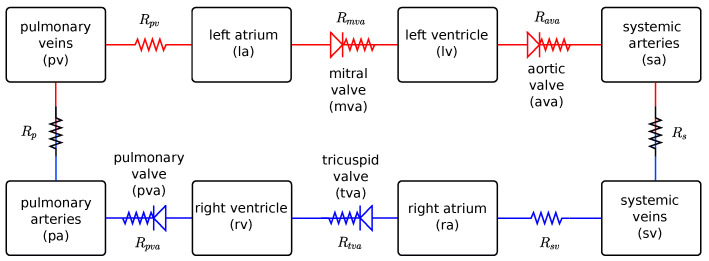
Schematic representation of the cardiovascular system model used in this study, based on a previous model [8].

The model involves a total of eight compartments, covering the four cardiac chambers (i.e., left and right atria and ventricles), as well as the pulmonary and systemic circulation represented by their respective arteries and veins, as illustrated in Figure 1.

In general, the model of the cardiovascular system, including cardiac, pulmonary and systemic circulation, can be described as:

(1) $$\begin{array}{cc} \frac{dV_{s,i}}{dt}\; & =q_{i-1}-q_{i}, \end{array}$$

(2) $$\begin{array}{cc} q_{i}\; & =\frac{p_{i}-p_{i+1}}{R_{i}}, \end{array}$$

(3) $$\begin{array}{cc} V_{s,i}\; & =V_{i}-V_{un,i}=C_{i}\left(p_{i}-p_{i+1}\right), \end{array}$$

where the indices i−1, *i* and i+1 refer to the previous, current and next compartments of the system, $V_{s,i}$ represents the stressed volume (the circulating volume) of the vessel/chamber, and $V_{un,i}$ is the unstressed volume (constant). In addition, $q_{i}$ is the blood flow, $p_{i}$ is the pressure, $R_{i}$ is the resistance between two compartments and $C_{i}$ is the complacency of a given compartment.

Considering the compartments and the diagram of the cardiovascular model used in this work, which is based on the work of Colunga et al. [8], as shown in Figure 1, we have the following ordinary differential equations that describe the volume variation of the compartments:

(4) $$\begin{array}{cc} \frac{dV_{pv}}{dt}\; & =q_{p}-q_{pv},\;\frac{dV_{la}}{dt}=q_{pv}-q_{mva},\;\frac{dV_{lv}}{dt}=q_{mva}-q_{ava},\;\frac{dV_{sa}}{dt}=q_{ava}-q_{s}, \end{array}$$

(5) $$\begin{array}{cc} \frac{dV_{pa}}{dt}\; & =q_{pva}-q_{p},\;\frac{dV_{rv}}{dt}=q_{tva}-q_{pva},\;\frac{dV_{ra}}{dt}=q_{sv}-q_{tva},\;\frac{dV_{sv}}{dt}=q_{s}-q_{sv}. \end{array}$$

The flows through the mitral (mva), aortic (ava), tricuspid (tva) and pulmonary (pva) valves are defined by the following equations:

(6) $$\begin{array}{cc} q_{mva}\; & =\left\{\begin{array}{cc} \frac{p_{la}-p_{lv}}{R_{mva}}, & \mathrm{if}\;p_{la}>p_{lv}\\ 0, & \mathrm{otherwise} \end{array}\right., \end{array}$$

(7) $$\begin{array}{cc} q_{ava}\; & =\left\{\begin{array}{cc} \frac{p_{lv}-p_{sa}}{R_{ava}}, & \mathrm{if}\;p_{lv}>p_{sa}\\ 0, & \mathrm{otherwise} \end{array}\right. \end{array}$$

(8) $$\begin{array}{cc} q_{tva}\; & =\left\{\begin{array}{cc} \frac{p_{ra}-p_{rv}}{R_{tva}}, & \mathrm{if}\;p_{la}>p_{lv}\\ 0, & \mathrm{otherwise} \end{array}\right., \end{array}$$

(9) $$\begin{array}{cc} q_{pva}\; & =\left\{\begin{array}{cc} \frac{p_{rv}-p_{pa}}{R_{pva}}, & \mathrm{if}\;p_{la}>p_{lv}\\ 0, & \mathrm{otherwise} \end{array}\right.. \end{array}$$

Cardiac dynamics are represented using time functions that relate the pressure in each cardiac chamber to elastance (as a function of time) and volume, as follows: $p_{i}\left(t\right)=E_{i}\left(\tilde{t}\right)V_{s,i}$, where i=ra,la,rv,lv and $E_{i}\left(\tilde{t}\right)$ is the time-dependent function describing the elastance of cardiac chamber *i*. Elastance functions for the ventricles and atria, along with additional details, can be found in the original references.

**Figure 2:**
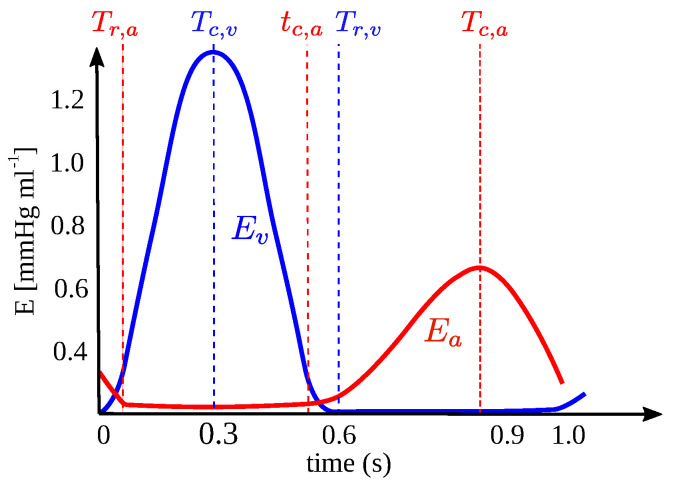
Schematic representation of the elastance function for the ventricular (blue) and atrial (red) chambers. Parameters corresponding to specific timings $T_{r,a}$, $T_{c,v}$, $t_{c,a}$, $T_{r,v}$ and $T_{c,a}$ are at their respective phases of the cardiac cycle.

The heart chambers i=ra,la,rv,lv denoting the left (*l*) and right (*r*) atria (*a*) and ventricles (*v*) are modeled using a time-varying elastance function $E_{i}\left(t\right)$, relating pressure and volume as follows:

(10) $$p_{i}\left(t\right)=E_{i}\left(\tilde{t}\right)V_{s,i},$$

where $\tilde{t}$ is a normalized time representing the time within the cardiac cycle. It is computed by $\tilde{t}=\mathrm{mod}\left(t,T\right)$, where *T* is the length of the cardiac cycle. The functions for the elastance of the ventricles and atria are illustrated schematically in Figure 2, and their mathematical expressions are detailed in Colunga et al. [8].

### 2.2. Immune System Model

The immune system (IS) model used to describe the dynamics during SARS-CoV-2 infection consists of fifteen ODEs. The model was originally proposed by Reis et al. [11] and has been qualitatively validated based on experimental data. The model represents the chain of events that occurs after SARS-CoV-2 infects immune cells. The hypothesis is that infected cells produce a high level of pro-inflammatory cytokines, which in turn can cause a cytokine storm (also known as *Cytokine Release Syndrome*—CRS). Cytokine storms have been associated, in addition to SARS-CoV-2 infection, with a wide variety of infectious and non-infectious diseases in recent decades.

The IS model is given by the following system of ODEs:

(11) $$\begin{array}{cc} \; & \frac{d}{dt}V=\pi_{v}V-k_{v1}VI_{gG}-k_{v1}VI_{gM}-k_{v2}VT_{ke}-k_{v3}VA_{pm},\\ \; & \frac{d}{dt}A_{p}=\alpha_{ap}\left(C+1\right)\left(A_{p0}-A_{p}\right)-\beta_{ap}A_{p}\frac{c_{ap1}V}{c_{ap2}+V},\\ \; & \frac{d}{dt}A_{pm}=\beta_{ap}A_{p}\frac{c_{ap1}V}{c_{ap2}+V}-\beta_{apm}A_{pm}V-\delta_{apm}A_{pm},\\ \; & \frac{d}{dt}I=\beta_{apm}A_{pm}V+\beta_{tke}T_{ke}V-\delta_{apm}I,\\ \; & \frac{d}{dt}T_{hn}=\alpha_{th}\left(T_{hn0}-T_{hn}\right)-\beta_{th}A_{pm}T_{hn},\\ \; & \frac{d}{dt}T_{he}=\beta_{th}A_{pm}T_{hn}+\pi_{th}A_{pm}T_{he}-\delta_{th}T_{he},\\ \; & \frac{d}{dt}T_{kn}=\alpha_{tk}\left(C+1\right)\left(T_{kn0}-T_{kn}\right)-\beta_{tk}\left(C+1\right)A_{pm}T_{kn},\\ \; & \frac{d}{dt}T_{ke}=\beta_{tk}\left(C+1\right)A_{pm}T_{kn}+\pi_{tk}A_{pm}T_{ke}-\beta_{tke}T_{ke}V-\delta_{tk}T_{ke},\\ \; & \frac{d}{dt}B=\alpha_{b}\left(B_{0}-B\right)+\pi_{b1}+VB+\pi_{b2}+T_{he}B-\beta_{ps}A_{pm}B-\beta_{pl}T_{he}B-\beta_{bm}T_{he}B,\\ \; & \frac{d}{dt}P_{s}=\beta_{ps}A_{pm}B-\delta_{ps}P_{s},\\ \; & \frac{d}{dt}P_{l}=\beta_{pl}T_{he}B-\delta_{pl}P_{l}+\gamma_{bm}B_{m},\\ \; & \frac{d}{dt}B_{m}=\beta_{bm}T_{he}B+\pi_{bm1}B_{m}\left(1-\frac{B_{m}}{\pi_{bm2}}-\gamma_{bm}B_{m}\right),\\ \; & \frac{d}{dt}I_{gM}=\pi_{ps}P_{s}-\delta_{am}I_{gM},\\ \; & \frac{d}{dt}I_{gG}=\pi_{pl}P_{l}-\delta_{ag}I_{gG},\\ \; & \frac{d}{dt}C=\pi_{c_{apm}}A_{pm}+\pi_{ci}I+\pi_{c_{tke}}T_{k}e-\delta_{c}C. \end{array}$$

Each of the ODEs of the immune system model represents the behavior of one of the populations involved in the disease and its fight, namely: virus (*V*), presenting cells (Ap), mature presenting cells ($A_{pm}$), immune cells infected by the SARS-CoV-2 virus (*I*), helper naïve T cells ($T_{hn}$), effector T helper cells ($T_{he}$), naïve killer T cells ($T_{kn}$), effector killer T cells ($T_{ke}$), B cells (*B*), short-lived plasma cells ($P_{s}$), long-lived plasma cells ($P_{l}$), memory B cells ($B_{m}$), IgM ($I_{gM}$) and IgG ($I_{gG}$) antibodies and cytokines (*C*). The rates and other parameters involved in the model are detailed in Reis et al. [11].

It is important to highlight that this particular IS model was developed to investigate two cases: case 1, composed of patients who survived the COVID-19 infection, and case 2, composed of patients who did not survive the disease. This approach allowed a comparative analysis between the two groups, specifically in relation to the levels of cytokines present in the immune system during infection with the SARS-CoV-2 virus. The results of the simulations also clarified why individuals who survived had lower levels of cytokines compared to those who did not survive. These substances play a vital role in the body’s immune response, and it is essential to regulate their function appropriately to effectively fight infection without causing additional damage to the body [11].

### 2.3. Effects of COVID-19 on the Cardiovascular System

The effects of COVID-19 on the CV system were modeled via changes in the following: (i) heart rate, (ii) resistance of the pulmonary veins and (iii) elastance of the four cardiac chambers. All changes were introduced through multiplicative factors that affect the original parameters of the CV model [12].

Changes in heart rate were implemented through a multiplicative factor $K_{T}$, which modifies the duration of the cardiac cycle and other parameters of the elastance functions appropriately. For instance, to change the heart rate from 60 bpm to 80 bpm, the model’s time constant values were multiplied by $K_{T}=0.75$.

Modifications in the contractility of the cardiac muscle were introduced through variations in the values of the maximum elastance parameters $E_{i,M}$, for i=ra, la, rv, lv, as follows:

(12) $$\begin{array}{c} E_{i,M}=K_{E}E_{i,M}^{ref} \end{array}$$

where $E_{i,M}^{ref}$ is the value of the maximum elastance of the cardiac chamber denoted by *i*.

For changes in pulmonary vein resistance, a multiplicative factor $K_{R}$ was introduced as follows:

(13) $$\begin{array}{c} R_{pv}=K_{R}R_{pv}^{ref}, \end{array}$$

where $R_{pv}^{ref}$ is the reference value for the normotensive individual.

It is important to note that in the original work of Dede et al. [12], the multiplying factors $K_{T}$, $K_{E}$ and $K_{R}$ were constants throughout the entire simulation. As will be shown in the following section, our coupling strategy for the CV and IS models considers these factors as a function of the cytokine concentrations *C* to describe the evolution of these effects during the infection.

### 2.4. Coupling of the Models

The objective of coupling the CV and IS models is to analyze the interactions between the immune system and the effects caused on the cardiovascular system during COVID-19 infection. For simplification purposes, the effects of COVID-19 on the cardiovascular system, are applied at specific points in time. We employed a simplified approach that considers only a one-way coupling, in which the IS model influences the cardiovascular CV model without feedback from the CV model to the IS. The simplification is based on the observation that the immune response to an infectious condition can be exaggerated, with greater than necessary production of pro-inflammatory cytokines, resulting in the phenomenon called cytokine storm. Therefore, for the coupling between the CV and IS models, cytokines were considered a fundamental component in describing the severity of the infectious condition and, consequently, the effects of COVID-19 on the CV model were calibrated based on the concentration of cytokines released by the immune system cells.

The coupling strategy involving $K_{T}$, $K_{EA}$ and $K_{RP}$ as functions of cytokines *C*, representing the effects of COVID-19 on the CV model, can be described as follows:

(14) $$\begin{array}{c} K_{T}\left(t\right)=1.0-\frac{C(t)}{C_{max}}0.3, \end{array}$$

(15) $$\begin{array}{c} K_{E}\left(t\right)=1.0-\frac{C(t)}{C_{max}}0.25, \end{array}$$

(16) $$\begin{array}{c} K_{R}\left(t\right)=1.0+\frac{C(t)}{C_{max}}1.75, \end{array}$$

where *C* represents the value of cytokine concentration, and $C_{max}$ is a parameter of the coupled model. The constants in the expressions are such that for $C=C_{max}$, the multipliers reach the reference values of the effects of COVID-19 on the CVS described in Dede et al. [12]. The parameter $C_{max}$ was considered a reference value for the maximum concentration of cytokines and was used to calibrate the model based on experimental data found in the literature [13]. The reference values used to describe the effects of COVID-19 on the CV are parameters that can be adjusted or tuned accordingly. The values scaled by $C/C_{max}$ were defined to ensure that when $C=C_{max}$, the baseline is modified in accordance with findings reported in the literature [12]. Specifically, at $C=C_{max}$, there is a 25% reduction in contractility and a 2.75-fold increase in pulmonary resistance relative to the baseline values.

### 2.5. Numerical Solution, Model Parameters and Initial Conditions

It is important to highlight that the time scales used in the two models are quite different: the cardiovascular system is evaluated in seconds, while the immune system is evaluated in days. Therefore, the approach adopted in this work is to simulate the coupled problem on the scale of days (referring to the IS) and, in certain steps, transfer the state of the IS model to the CV model. This procedure is carried out according to an interval given by $\Delta_{CV}$, that is, every $\Delta_{CV}$ days the cytokines from the IS model are transferred to the CV model. At this point, the CV model is solved for a certain number of cardiac cycles $N_{C}$ until reaching a steady state, where the effects on the CV are evaluated. For the studies of this work, we used $N_{C}=30$ cycles. For the coupled model, a total of $N_{CV}=24$ time windows for the coupling were considered, which resulted in a total of 120 days for the simulation.

The coupled CV+IS model is defined by the Equations (1)–(3) from the CV model and (11) from the IS model. The coupled model was numerically solved as follows: the cardiovascular model was computed using the LSODA method via the odeint interface from the SciPy library, while the immune system model was solved using the RK45 method through the solve_ivp interface, also available in SciPy [14]. We used a time step size equal to Δt=0.001 s for the numerical integration of the CV model, and Δt=1 days for the IS model. For the coupling, we adopted in this work as $\Delta_{CV}=2$ days, which means that every 2 days information from the IS model is transferred to the CV model.

Table A1 and Table A2 summarize the parameter values used for the CV and IS components of the coupled model, respectively. Table A3 lists the initial conditions for all variables of the coupled model. It is important to note that the CV parameter values and initial conditions were adjusted based on clinical information from a normotensive individual, as documented by Colunga et al. [8].

The parameters and initial conditions of the IS model are available in Reis et al. [11] (Tables 5 and 6), and were also based on experimental data. It is important to highlight that the IS model parameters were fitted for two scenarios, which are also explored in this work: survivors and non-survivors. The IS model parameters were calibrated using available cohort data on viremia, IgG, IgM and cytokines for both survivors and non-survivors. To achieve this, the discrepancy between the model and the observed data was minimized using a weighted least-squares approach, employing the differential evolution method. A detailed description is provided in Reis et al. [11].

### 2.6. Uncertainty Quantification

In this work, we considered values for $C_{max}$ as those reported in the literature [13]. Due to the biological variability, the values reported by Cabaro et al. [13] for the levels of cytokines in a mild COVID-19 scenario are within a range of [8.07,26.98] pg/mL. The data are also consistent with other values reported in the literature [15,16,17,18]. Therefore, to evaluate the impacts of this parameter within this range, we conducted an uncertainty propagation considering $C_{max}$ as a random variable uniformly distributed in its physiological range. So, we considered

(17) $$\begin{array}{c} C_{max}∼U\left(8.07,26.98\right), \end{array}$$

where *U* represents the uniform distribution.

To carry out this uncertainty quantification, we used the classical Monte Carlo method with a total number of 1000 realizations (executions of the coupled model) considering only $C_{max}$ as an uncertain input parameter. Then, relevant quantities of interest in the model, such as the pressure–volume loop (PV-loop) and the ejection fraction (EF), were evaluated statistically through the mean value and prediction intervals obtained by specific percentiles of the outputs. For more details on uncertainty quantification for biomedical applications, see the review by Eck et al. [19].

### 2.7. Pressure–Volume Loop and Ejection Fraction

Below, we describe two key metrics used to evaluate cardiac function. A widely used method for assessing the cardiovascular system and evaluating ventricular function involves diagrams that plot the relationship between pressure within the ventricular cavities and their corresponding volumes. These diagrams, commonly referred to as *PV loops*, offer crucial insights into ventricular performance and dynamics, serving as a fundamental tool for understanding cardiac function [20].

Ejection fraction (EF) is another key measure of cardiac performance defined as the percentage of blood ejected from a ventricle during systole relative to its end-diastolic volume. The ejection fraction is given by $EF=\frac{EDV-ESV}{EDV}$, where EDV and ESV denote the end-diastolic and end-systolic volumes, respectively. Ejection fraction is crucial for assessing ventricular efficiency and diagnosing conditions such as heart failure, as it reflects the heart’s ability to pump blood effectively. *EF* is most commonly associated with the left ventricle, as it reflects the efficiency of this chamber in pumping oxygenated blood to the systemic circulation. Normal values for EF are typically between 50% and 70%. It is worth noting that these values may vary according to age [21], sex [22] and other conditions, such as during inflammation [23].

## 3. Results

This section presents the numerical results obtained through the simulation of the coupled model that represents the interaction between the immune system and the cardiovascular system in the context of SARS-CoV-2 infection. Through the analysis of simulations carried out for different scenarios (survivors and non-survivors), the interactions between the two systems are described. We denote by case 1 the scenario simulated with the parameters adjusted for the survivors and case 2 the scenario simulated for the non-survivors.

### 3.1. Immune System and Cardiovascular Models

We begin by presenting an illustrative simulation of the coupled CV+IS model using the baseline parameters. First, we present the results of each model component, and then we detail how one model influences the other.

**Figure 3:**
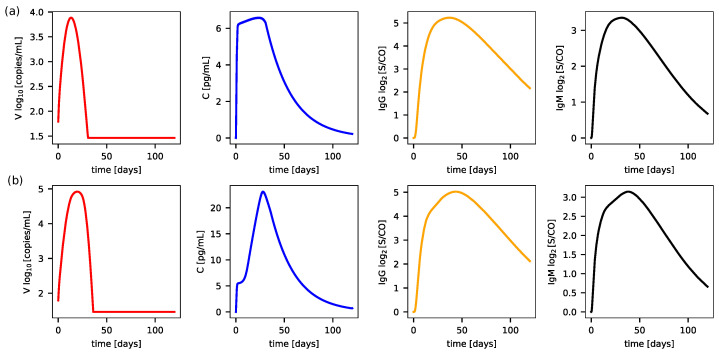
Evolution of the immune system variables *V*, *C*, $I_{gG}$ e $I_{gM}$ for the (**a**) survivors case 1 and for the (**b**) non-survivors case 2.

Figure 3 presents the results of the simulations carried out for four variables of the IS model for cases 1 and 2 (survivors and non-survivors) fixing $C_{max}=12$ pg/mL. The variables represent the viremia *V*, the cytokine concentration *C* (used in coupling) and also the variables $I_{gG}$ and $I_{gM}$.

It can be seen in the evolution of the cytokine concentration in case 1 at the top panel (a) of Figure 3, that cytokines increase rapidly until reaching high values and maintain this trend through a plateau phase, reaching levels of cytokines about 6 pg/mL, and then decay back to equilibrium at the end of the simulation of 120 days. The analysis of the evolution of viremia allows us to conclude that, in this case, the IS was efficient and effectively fought the virus until its complete elimination. For case 2, shown in the bottom panel (b) of Figure 3, related to the non-survivors, the results follow the same trends. However, it can be observed that the concentration of the cytokines reaches much higher values than in the previous case, as does the amount of virus.

**Figure 4:**
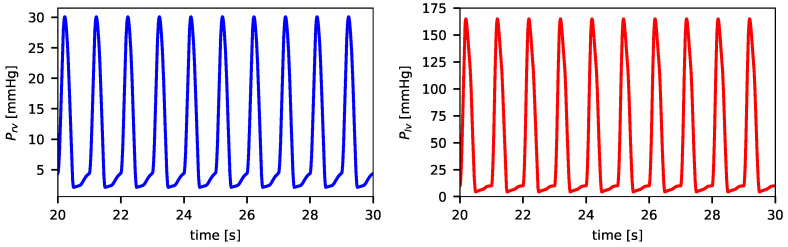
Evolution of the cardiovascular variables $P_{rv}$ and $P_{lv}$ describing right and left ventricle pressures, respectively, at the beginning of the coupled simulation of the survivors case 1.

Figure 4 illustrates the typical evolution of right and left ventricular pressures, $P_{rv}$ and $P_{lv}$, for the simulation of survivors (Case 1). The data shown correspond to the final ten cardiac cycles at the onset of the coupled simulation, where it is clear that the quantities reached a steady state.

### 3.2. Coupled Model

**Figure 5:**
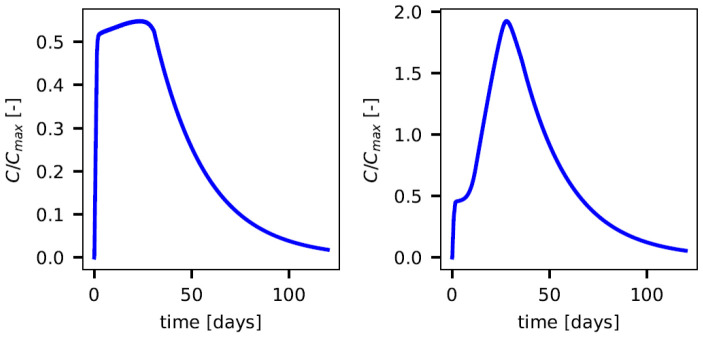
Link for the coupling of the CV and IS models: evolution of $C/C_{max}$ for case 1 (**left**) and case 2 (**right**), where $C_{max}$ was fixed at the value of 12 pg/mL.

Next, we present the evolution of the key component linking the IS model to the cardiovascular model, which is the evolution of the cytokines scaled by the $C_{max}$ parameter. Here, we considered $C_{max}=12$ pg/mL fixed, while in the next studies, it was considered a random variable. Figure 5 shows the evolution of the coupling variable between the IS and CV model for both survivors and non-survivors.

**Figure 6:**
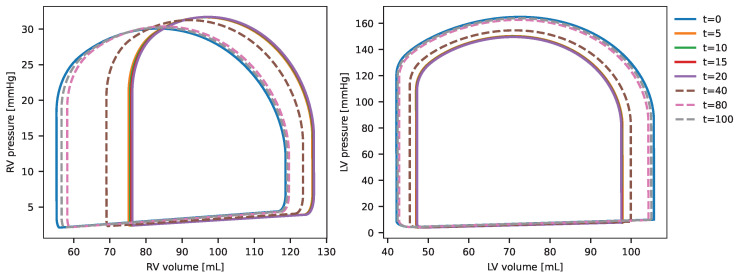
PV-loop evolution of left and right ventricles for simulation case 1 (survivors) in the coupled model.

The results of the coupled model, illustrated by the PV-loops of the left and right ventricles for the survivor case (case 1), are presented in Figure 6. The graphs depict the patient’s PV-loop under normal conditions prior to infection t=0 and demonstrate how the effects of infection’s progression, coupled with immune system activation, influence the cardiovascular system through the cytokine effects described earlier. These impacts are evident in the temporal changes of the PV-loops. The peak effect of cytokines on the cardiovascular system occurs between t=2 and t=20 days, marked by a reduction in ejection fraction. Subsequently, as the immune system combats the infection, the cardiovascular system gradually recovers, with normal function restored from t=80 days. Notably, the dynamics are most pronounced at the simulation’s onset, with significant PV-loop alterations occurring within the first days due to the strong interaction between the cardiovascular and immune system models.

**Figure 7:**
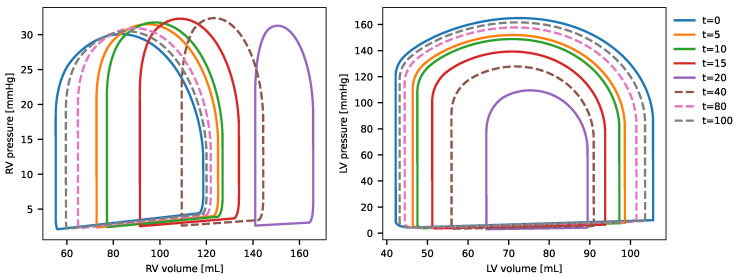
PV-loop evolution of left and right ventricles for simulation case 2 (survivors) in the coupled model.

Figure 7 illustrates the evolution of the PV-loops for the right and left ventricles in the non-survivor case (case 2). The dynamics unfold more gradually compared to case 1, with incremental changes in the PV-loops observed at each time point depicted in the figure. The maximal impact of inflammation, driven by immune system activation of cytokines, occurs around t=20 days, where the PV-loops and EF are notably altered from the baseline (t=0), reflecting the effects of the infection. Following this peak, the influence of cytokines on the cardiovascular system diminishes, and the model shows a recovery in the PV-loops, approaching baseline values as the system begins to restore its original function.

When comparing the PV-loops and ejection fraction between survivor and non-survivor cases, the changes observed in the survivor case (case 1) are less pronounced than in the non-survivor case (case 2). In case 2, significant alterations in both the PV-loop and EF are evident, reflecting a more substantial impact of inflammation. This aligns with the fact that the parameters of the IS model in case 2 were calibrated from data of non-survivor individuals, who experienced a stronger immune response and more severe inflammatory effects.

### 3.3. Uncertainty Quantification

Next, we evaluated the coupled model treating $C_{max}$ as a random variable constrained within its reported physiological range. The results of this uncertainty propagation are presented as the 90% prediction interval (encompassing the 5th to 95th percentiles) for the PV-loops, along with the evolution of the ejection fraction over time obtained from the simulations.

**Figure 8:**
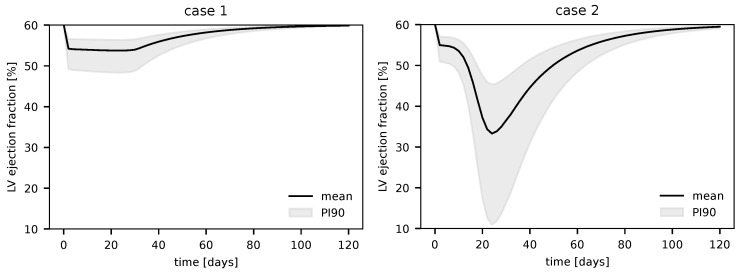
Uncertainty propagation for the left ventricle ejection fraction for cases 1 and 2. The solid line represents the mean ejection fraction, while the shaded region is the 90% prediction interval (PI90).

Figure 8 illustrates the evolution of the ejection fraction calculated at the conclusion of the $N_{C}$ cycles for each coupling step between the IS and CV models, using $\Delta_{CV}=2$ days for the simulations. It can be observed that in both cases, the EF initially declines during the inflammation phase and subsequently recovers to its baseline value. This behavior highlights the influence of cytokine transients on the CV model response and the shape of the left ventricular EF curve.

Notably, in case 2 (non-survivors), the EF values fall to markedly low levels, well below the normal physiological range, whereas in case 1 (survivors), the EF remains within normal limits (about 50%) even when accounting for uncertainty. For example, in case 2, the left ventricular EF decreases to as low as 20%, indicating severe cardiac dysfunction. Such impairment is strongly associated with increased hospitalization and mortality rates [24].

**Figure 9:**
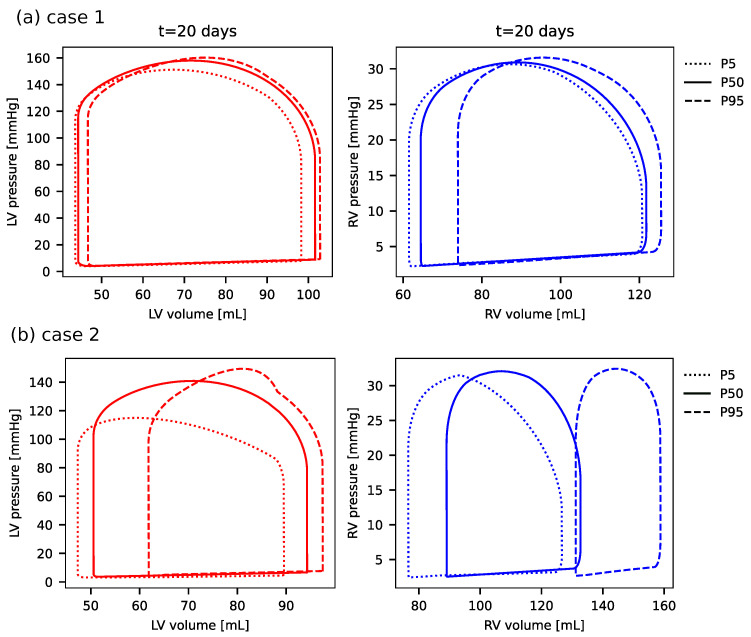
Uncertainties in the left and right ventricle pressure–volume loops for case 1 (**a**) and case 2 (**b**) at t=20 days of simulation of the coupled model. The dotted line represents the 5th percentile, the solid line is the 50th percentile and the dashed line is the 95th percentile.

Figure 9 shows the uncertainty quantification for the pressure–volume loops of the RV and LV, as measured at t=20 days for both cases 1 and 2 corresponding to survivors (a) and non-survivors (b), respectively. The uncertainty is presented using the 5th, 50th and 95th percentiles of the pressure–volume loops from all samples obtained during the analysis. Firstly, one can clearly observe that the uncertainties for the survivor’s case are significantly smaller than those for non-survivors. Secondly, one can notice that although the uncertainties for the LV pressure–volume loop are non-negligible, the ones for the RV are much higher.

### 3.4. Computational Performance

In this section, we report the total elapsed time for a single simulation of the coupled CV+IS model, as well as for the complete Monte Carlo analysis conducted for uncertainty quantification. All simulations were performed on a Dell Latitude 3440 laptop equipped with an Intel Core i5-1235U processor and 8 GB of RAM.

The total elapsed time for a single run of the coupled CV+IS model, using a time step of Δt=0.001 s for the CV model, Δt=1 day for the IS model and a coupling interval of $\Delta_{CV}=2$ days, was about 15 s. The total elapsed time for the Monte Carlo analysis using 1000 samples of the $C_{max}$ parameter was about 13,200 s.

## 4. Discussion

### 4.1. On the Immune System Response and Cytokine Release

The immune system’s response to SARS-CoV-2 infection involves a cascade of events that can lead to either protective or pathological outcomes. In our coupled model simulations, the cytokine response emerged as a critical determinant of the clinical trajectory of COVID-19, particularly due to its impact on cardiovascular function.

In the case of survivors (case 1, Figure 3), cytokine levels rose rapidly during the initial phases of infection, peaking before gradually declining. This controlled cytokine production facilitated viral clearance without causing excessive systemic damage. Conversely, in non-survivors (case 2, Figure 3b), cytokine levels exhibited an initial peak in the early days of infection, followed by a significantly elevated second peak. This excessive response, often referred to as a cytokine storm, exacerbated systemic inflammation and was strongly associated with severe cardiovascular dysfunction.

Cytokines, such as interleukins (e.g., IL-6) and tumor necrosis factor-alpha (TNF-α), play a dual role during infection. On the one hand, they activate immune cells and coordinate the antiviral response; on the other, their overproduction can result in endothelial dysfunction, increased vascular permeability and tissue damage. Our model’s simulation of these dynamics highlights the critical balance required in cytokine regulation to prevent complications such as myocardial injury, arrhythmia and thromboembolic events—complications observed to be more pronounced in non-survivors (case 2).

Finally, Figure 3 shows that antibody production and viral elimination are not significantly affected by increased cytokine production. Both survivors and non-survivors exhibited similar peak levels of IgM and IgG, with only a slight increase in viral load concentration in non-survivors compared to survivors.

### 4.2. Effects on the Cardiovascular System

The simulations of survivors (case 1), while influenced by cytokine, exhibited an ejection fraction and ventricular response indicative of inflammation but ultimately demonstrated an overall normal cardiac function with values still in the normal ranges [25,26], as can be noticed in Figure 6, Figure 8 and Figure 9a.

The effects of reduced contractility during COVID-19 infection were modeled by the reduction in $K_{EA}$ used to decrease the value of the four active elastances. For case 1, this change resulted in a maximal reduction of 25% of the baseline EF, whereas for case 2, it resulted in drastic changes in the ejection fraction of up to 400%, as shown by the lower limits of uncertainty in Figure 8.

In addition to the left ventricular function, the right ventricular function was also markedly impacted by COVID-19-induced alterations in the simulations of the coupled model. More specifically, the RV EF reduced about 50% for case 1, while for the non-survivors case, a 5-fold reduction was observed. One can note that this clearly shows a significant reduction in the RV EF when compared to the LV EF.

The observed reductions in ejection fraction (EF) of 25% in the left ventricle (LV) and 50% in the right ventricle (RV) for the survivor case (Case 1) are consistent with post-COVID-19 clinical findings. Specifically, Koutsiaris et al. [27] reported an average hemodynamic reduction of 45% in conjunctival microcirculation, while Zharkikh et al. [28] documented a 29% reduction in skin microcirculation. Furthermore, the EF reduction aligns with a recently proposed pathophysiological mechanism of blood supply reduction in post-COVID-19 patients, based on data from 634 individuals [29]. These cohort studies clearly show that COVID-19 affects the cardiovascular system, causing endothelial dysfunction, microvascular damage and increased heart failure risk.

The increased pulmonary resistance, introduced via the $K_{RP}$ factor in the coupled model, led to an approximately 10% rise in the RV maximal pressure in both cases. Compared to other cardiovascular models in the literature, such as those presented by Dede et al. [12], this effect is slightly less pronounced. However, it is important to note that the modeling approaches for the cardiovascular system differ between these studies.

Finally, it is essential to emphasize that the simulations for case 2, representing non-survivors of COVID-19, reported a left ventricular ejection fraction (LVEF) as low as 15% in the uncertainty propagation study. According to the literature, an LVEF below 35% is typically associated with a poor prognosis and an increased risk of mortality. Given that the data used for the immune system IS model in these simulations were derived from a cohort of non-survivors, we consider the model to have effectively captured and satisfactorily represented this clinical scenario.

## 5. Conclusions

This study proposed a coupling model between the immune system and the cardiovascular system to analyze the effects of infections, such as COVID-19, on cardiovascular dynamics. The coupled model integrates two previously published and experimentally calibrated models. The CV model employs a lumped-parameter representation of the cardiovascular system, comprising eight compartments, while the IS model represented by a system of fifteen ordinary differential equations, simulates the immune response to SARS-CoV-2 and its response with an elevated production of inflammatory cytokines.

To accommodate the differing time scales of the two systems, a unidirectional coupling strategy was adopted, with information flowing from the IS model to the CV model. The CV model is resolved only within specific time windows, consistent with this approach. Both models were previously validated against experimental data. In this study, the coupling is mediated by cytokine concentrations, which, when elevated, can adversely affect cardiovascular function.

The COVID-19 pandemic has underscored the importance of understanding the interplay between the cardiovascular and immune systems during viral infections. SARS-CoV-2 enters human cells via the ACE-2 enzyme, initiating a cascade of immune responses. This study presents a coupled mathematical model that integrates the cardiovascular system (CVS) and immune system (IS), capturing their complex interactions during infection. The CVS model, based on ordinary differential equations, describes heart dynamics and pulmonary and systemic circulation, while the IS model simulates immune responses to SARS-CoV-2, including immune cell interactions and cytokine production. A coupling strategy transfers information from the IS to the CVS at specific intervals, enabling the exploration of immune-driven cardiovascular effects.

Numerical simulations examined how these interactions influence infection severity and recovery. The coupled model accurately replicated the evolution of cardiac function in survivors and non-survivors of COVID-19. Survivors exhibited a left ventricular ejection fraction (LVEF) reduction of up to 25% while remaining within normal limits, whereas non-survivors showed a severe 4-fold decline, indicative of myocardial dysfunction. Similarly, the right ventricular ejection fraction (RV EF) decreased by approximately 50% in survivors but underwent a drastic 5-fold reduction in non-survivors, reflecting the severe impact on right ventricular function. The numerical results align with experimental findings [3,5,6,27,28,29] that describe the impacts of COVID-19 on the cardiovascular system. These findings highlight the model’s capacity to distinguish differential cardiac dysfunction across clinical outcomes and its potential to enhance our understanding of COVID-19 pathophysiology.

## Data Availability

The original contributions presented in this study are included in this article. Further inquiries can be directed to the corresponding authors.

## Appendix A

This appendix presents the parameters and initial conditions of the CV and IS models.

**Table A1:** Reference parameters for the cardiovascular (CV) component of the coupled model.

Parameter	Value	Unit	Parameter	Value	Unit
$R_{ava}$	0.1291	$\mathrm{mmHg}\;s\;{\mathrm{mL}}^{-1}$	$R_{mva}$	0.0258	$\mathrm{mmHg}\;s\;{\mathrm{mL}}^{-1}$
$R_{pva}$	0.0387	$\mathrm{mmHg}\;s\;{\mathrm{mL}}^{-1}$	$R_{tva}$	0.0517	$\mathrm{mmHg}\;s\;{\mathrm{mL}}^{-1}$
$R_{sv}$	0.1550	$\mathrm{mmHg}\;s\;{\mathrm{mL}}^{-1}$	$R_{pv}$	0.0258	$\mathrm{mmHg}\;s\;{\mathrm{mL}}^{-1}$
$R_{s}$	1.3560	$\mathrm{mmHg}\;s\;{\mathrm{mL}}^{-1}$	$R_{p}$	0.0646	$\mathrm{mmHg}\;s\;{\mathrm{mL}}^{-1}$
$C_{sa}$	1.3590	$\mathrm{mL}\;{\mathrm{mmHg}}^{-1}$	$C_{pa}$	5.5753	$\mathrm{mL}\;{\mathrm{mmHg}}^{-1}$
$C_{sv}$	15.0998	$\mathrm{mL}\;{\mathrm{mmHg}}^{-1}$	$C_{pv}$	5.6218	$\mathrm{mL}\;{\mathrm{mmHg}}^{-1}$
$E_{ra,M}$	0.2333	$\mathrm{mmHg}\;{\mathrm{mL}}^{-1}$	$E_{ra,m}$	0.0464	$\mathrm{mmHg}\;{\mathrm{mL}}^{-1}$
$E_{la,M}$	0.3500	$\mathrm{mmHg}\;{\mathrm{mL}}^{-1}$	0.1237	$\mathrm{mmHg}\;{\mathrm{mL}}^{-1}$	
$E_{rv,M}$	0.4239	$\mathrm{mmHg}\;{\mathrm{mL}}^{-1}$	$E_{rv,m}$	0.0377	$\mathrm{mmHg}\;{\mathrm{mL}}^{-1}$
$E_{lv,M}$	3.0616	$\mathrm{mmHg}\;{\mathrm{mL}}^{-1}$	$E_{lv,m}$	0.0942	$\mathrm{mmHg}\;{\mathrm{mL}}^{-1}$
$\tau_{ra,c}$	0.6500	s	$\tau_{la,c}$	0.6825	s
$T_{ra,c}$	0.8500	s	$T_{la,c}$	0.8500	s
$T_{ra,r}$	0.2500	s	$T_{la,r}$	0.2525	s
$T_{rv,c}$	0.3000	s	$T_{lv,c}$	0.2850	s
$T_{rv,r}$	0.5000	s	$T_{lv,r}$	0.5000	s

**Table A2:** Reference parameters for the immune system (IS) component of the coupled model (model parameter values that fit survivors’ data).

Parameter	Value	Unit
$\pi_{v}$	$1.47\times 10^{0}$	day^−1^
$k_{v1}$	$9.82\times 10^{-3}$	day^−1^ mIU/mL^−1^
$k_{v2}$	$6.10\times 10^{-5}$	day^−1^ cells/mL^−1^
$k_{v3}$	$6.45\times 10^{-2}$	day^−1^ cells/mL^−1^
$\alpha_{ap}$	$1.0\times 10^{0}$	day^−1^ pg/mL^−1^
$\beta_{ap}$	$1.79\times 10^{-1}$	day^−1^ copies/mL^−1^
$c_{ap1}$	$8.0\times 10^{0}$	copies/mL
$c_{ap2}$	$8.08\times 10^{6}$	copies/mL
$\delta_{apm}$	$4.0\times 10^{-2}$	day^−1^
$\beta_{apm}$	$1.33\times 10^{-2}$	day^−1^ copies/mL^−1^
$\beta_{tke}$	$3.5\times 10^{-6}$	day^−1^ copies/mL^−1^
$\alpha_{th}$	$2.17\times 10^{-4}$	day^−1^
$\beta_{th}$	$1.8\times 10^{-5}$	day^−1^ cells/mL^−1^
$\pi_{th}$	$1.0\times 10^{-8}$	day^−1^ cells/mL^−1^
$\delta_{th}$	$3.0\times 10^{-1}$	day^−1^
$\alpha_{tk}$	$1.0\times 10^{0}$	day^−1^ pg/mL^−1^
$\beta_{tk}$	$1.43\times 10^{-5}$	day^−1^ pg/mL^−1^ cells/mL^−1^
$\pi_{tk}$	$1.0\times 10^{-8}$	day^−1^ cells/mL^−1^
$\delta_{tk}$	$3\times 10^{-2}$	day^−1^
$\alpha_{b}$	$3.58\times 10^{0}$	day^−1^
$\pi_{b1}$	$8.98\times 10^{-5}$	day^−1^ copies/mL^−1^
$\pi_{b2}$	$1.27\times 10^{-8}$	day^−1^ cells/mL^−1^
$\beta_{ps}$	$6.0\times 10^{-6}$	day^−1^ cells/mL^−1^
$\beta_{pl}$	$5.0\times 10^{-6}$	day^−1^ cells/mL^−1^
$\beta_{bm}$	$1.0\times 10^{-6}$	day^−1^ cells/mL^−1^
$\delta_{ps}$	$2.5\times 10^{0}$	day^−1^
$\delta_{pl}$	$3.5\times 10^{-1}$	day^−1^
$\gamma_{bm}$	$9.75\times 10^{-4}$	day^−1^
$\pi_{bm1}$	$1.0\times 10^{-5}$	day^−1^
$\pi_{bm2}$	$2.5\times 10^{3}$	cells/mL
$\pi_{ps}$	$8.7\times 10^{-2}$	day^−1^ cells/mL^−1^ S/CO
$\pi_{pl}$	$1.0\times 10^{-3}$	day^−1^ cells/mL^−1^ S/CO
$\delta_{am}$	$7.0\times 10^{-2}$	day^−1^
$\delta_{ag}$	$7.0\times 10^{-2}$	day^−1^
$\pi_{c_{apm}}$	$3.28\times 10^{2}$	day^−1^ cells/mL^−1^ pg/mL
$\pi_{ci}$	$6.44\times 10^{-3}$	day^−1^ cells/mL^−1^ pg/mL
$\pi_{c_{tke}}$	$1.78\times 10^{-2}$	day^−1^ cells/mL^−1^ pg/mL
$\delta_{c}$	$7.04\times 10^{2}$	day^−1^

**Table A3:** Initial conditions of the coupled cardiovascular (CV) and immune system (IS) model.

Parameter	Value	Unit	Parameter	Value	Unit
$V_{sa}$	163.078	mL	$V_{sv}$	226.498	mL
$V_{ra}$	34.287	mL	$V_{rv}$	106.153	mL
$V_{pa}$	83.630	mL	$V_{pv}$	56.218	mL
$V_{la}$	34.287	mL	$V_{lv}$	106.153	mL
*V*	61.0	copies/mL	$A_{p}$	$1.0\times 10^{6}$	cell/mL
$A_{pm}$	0.0	cell/mL	*I*	0.0	cells/mL
$T_{hn}$	$1.0\times 10^{6}$	cells/mL	$T_{hE}$	0.0	cells/mL
$T_{kN}$	$5.0\times 10^{5}$	cells/mL	$T_{kE}$	0.0	cells/mL
*B*	$2.5\times 10^{5}$	cells/mL	$P_{s}$	0.0	cells/mL
$P_{l}$	0.0	cells/mL	$B_{m}$	0.0	cells/mL
$I_{gM}$	0.0	S/CO	$I_{gG}$	0.0	S/CO
*C*	0.0	pg/mL			

## References

[1] World Health Organization (2020). World Health Organization Coronavirus Disease (COVID-19) Outbreak.

[2] Scholz J.R., Lopes M.A.C.Q., Saraiva J.F.K., Colombo F.C. (2020). COVID-19, Sistema Renina-Angiotensina, Enzima Conversora da Angiotensina 2 e Nicotina: Qual a Inter-Relação?. Arq. Bras. Cardiol..

[3] Zheng Y.Y., Ma Y.T., Zhang J.Y., Xie X. (2020). COVID-19 and the cardiovascular system. Nat. Rev. Cardiol..

[4] Tajbakhsh A., Gheibi Hayat S.M., Taghizadeh H., Akbari A., inabadi M., Savardashtaki A., Johnston T.P., Sahebkar A. (2020). COVID-19 and cardiac injury: Clinical manifestations, biomarkers, mechanisms, diagnosis, treatment, and follow up. Expert Rev. Anti-Infect. Ther..

[5] Guzik T.J., Mohiddin S.A., Dimarco A., Patel V., Savvatis K., Marelli-Berg F.M., Madhur M.S., Tomaszewski M., Maffia P., D’Acquisto F. (2020). COVID-19 and the cardiovascular system: Implications for risk assessment, diagnosis, and treatment options. Cardiovasc. Res..

[6] Gupta A., Madhavan M.V., Sehgal K., Nair N., Mahajan S., Sehrawat T.S., Bikdeli B., Ahluwalia N., Ausiello J.C., Wan E.Y. (2020). Extrapulmonary manifestations of COVID-19. Nat. Med..

[7] Lourenço W.d.J., Reis R.F., Ruiz-Baier R., Rocha B.M., dos Santos R.W., Lobosco M. (2022). A Poroelastic Approach for Modelling Myocardial Oedema in Acute Myocarditis. Front. Physiol..

[8] Colunga A.L., Colebank M.J., Program R., Olufsen M.S. (2023). Parameter inference in a computational model of haemodynamics in pulmonary hypertension. J. R. Soc. Interface.

[9] Marquis A.D., Arnold A., Dean-Bernhoft C., Carlson B.E., Olufsen M.S. (2018). Practical identifiability and uncertainty quantification of a pulsatile cardiovascular model. Math. Biosci..

[10] Shi Y., Lawford P., Hose R. (2011). Review of zero-D and 1-D models of blood flow in the cardiovascular system. Biomed. Eng. Online.

[11] Reis R.F., Pigozzo A.B., Bonin C.R.B., Quintela B.d.M., Pompei L.T., Vieira A.C., Xavier M.P., Weber dos Santos R., Lobosco M., Silva L.D.L.E. (2021). A validated mathematical model of the cytokine release syndrome in severe COVID-19. Front. Mol. Biosci..

[12] Dedè L., Regazzoni F., Vergara C., Zunino P., Guglielmo M., Scrofani R., Fusini L., Cogliati C., Pontone G., Quarteroni A. (2021). Modeling the cardiac response to hemodynamic changes associated with COVID-19: A computational study. Math. Biosci. Eng..

[13] Cabaro S., D’Esposito V., Di Matola T., Sale S., Cennamo M., Terracciano D., Parisi V., Oriente F., Portella G., Beguinot F. (2021). Cytokine signature and COVID-19 prediction models in the two waves of pandemics. Sci. Rep..

[14] Virtanen P., Gommers R., Oliphant T.E., Haberland M., Reddy T., Cournapeau D., Burovski E., Peterson P., Weckesser W., Bright J. (2020). SciPy 1.0: Fundamental algorithms for scientific computing in Python. Nat. Methods.

[15] Biancotto A., Wank A., Perl S., Cook W., Olnes M.J., Dagur P.K., Fuchs J.C., Langweiler M., Wang E., McCoy J.P. (2013). Baseline levels and temporal stability of 27 multiplexed serum cytokine concentrations in healthy subjects. PLoS ONE.

[16] Ebihara T., Matsumoto H., Matsubara T., Togami Y., Nakao S., Matsuura H., Kojima T., Sugihara F., Okuzaki D., Hirata H. (2022). Cytokine elevation in severe COVID-19 from longitudinal proteomics analysis: Comparison with sepsis. Front. Immunol..

[17] Leisman D.E., Ronner L., Pinotti R., Taylor M.D., Sinha P., Calfee C.S., Hirayama A.V., Mastroiani F., Turtle C.J., Harhay M.O. (2020). Cytokine elevation in severe and critical COVID-19: A rapid systematic review, meta-analysis, and comparison with other inflammatory syndromes. Lancet Respir. Med..

[18] Sanchez-de Prada L., Gorgojo-Galindo O., Fierro I., Martínez-García A.M., de Quintana G.S.L., Gutiérrez-Bustillo R., Pelaez-Jareño M.T., Alvarez-Fuente E., Gomez-Sanchez E., Tamayo E. (2022). Time evolution of cytokine profiles associated with mortality in COVID-19 hospitalized patients. Front. Immunol..

[19] Eck V.G., Donders W.P., Sturdy J., Feinberg J., Delhaas T., Hellevik L.R., Huberts W. (2016). A guide to uncertainty quantification and sensitivity analysis for cardiovascular applications. Int. J. Numer. Methods Biomed. Eng..

[20] Burkhoff D. (2013). Pressure-volume loops in clinical research: A contemporary view. J. Am. Coll. Cardiol..

[21] Lakatta E. (1990). Changes in cardiovascular function with aging. Eur. Heart J..

[22] Kaur G., Lau E. (2022). Sex differences in heart failure with preserved ejection fraction: From traditional risk factors to sex-specific risk factors. Women’s Health.

[23] Pugliese N.R., Pellicori P., Filidei F., De Biase N., Maffia P., Guzik T.J., Masi S., Taddei S., Cleland J.G. (2022). Inflammatory pathways in heart failure with preserved left ventricular ejection fraction: Implications for future interventions. Cardiovasc. Res..

[24] Angaran P., Dorian P., Ha A.C., Thavendiranathan P., Tsang W., Leong-Poi H., Woo A., Dias B., Wang X., Austin P.C. (2020). Association of left ventricular ejection fraction with mortality and hospitalizations. J. Am. Soc. Echocardiogr..

[25] Cain P.A., Ahl R., Hedstrom E., Ugander M., Allansdotter-Johnsson A., Friberg P., Arheden H. (2009). Age and gender specific normal values of left ventricular mass, volume and function for gradient echo magnetic resonance imaging: A cross sectional study. BMC Med. Imaging.

[26] Maceira A.M., Prasad S.K., Khan M., Pennell D.J. (2006). Reference right ventricular systolic and diastolic function normalized to age, gender and body surface area from steady-state free precession cardiovascular magnetic resonance. Eur. Heart J..

[27] Koutsiaris A.G., Riri K., Boutlas S., Panagiotou T.N., Kotoula M., Daniil Z., Tsironi E.E. (2022). COVID-19 hemodynamic and thrombotic effect on the eye microcirculation after hospitalization: A quantitative case-control study. Clin. Hemorheol. Microcirc..

[28] Zharkikh E.V., Loktionova Y.I., Fedorovich A.A., Gorshkov A.Y., Dunaev A.V. (2023). Assessment of blood microcirculation changes after COVID-19 using wearable laser Doppler flowmetry. Diagnostics.

[29] Koutsiaris A.G. (2024). A Blood Supply Pathophysiological Microcirculatory Mechanism for Long COVID. Life.

